# Conservatism in Modern Therapeutics1Read at the thirty-ninth annual meeting of the Medical Association of Central New York at Syracuse, October 16, 1906.

**Published:** 1907-06

**Authors:** Milton E. Gregg

**Affiliations:** Elbridge, N. Y.


### Conservatism in Modern Therapeutics.[1]

By MILTON E. GREGG, M.D., Elbridge, N. Y.

THERAPEUTICS is perhaps the highest and most skilful of all the medical arts. She comes to us the child of the selfsacrificing and oftentimes unremunerated toil of the centuries gone by. Out of the clouds and thick darkness of the past she emerges as an angel of light with a glorious mission, the alleviation of human suffering and the saving of human life. Should we not guard her well? Should we not watch over her with jealous eye promptly repelling any influence that tends to lessen her power for good? That such influences are at work is evident to the careful observer and they demand our earnest consideration. I shall briefly discuss a fact, a principle, and a duty.

That this is an age of new things in therapeutics is a fact requiring little demonstration. In pharmacy we have a multitudinous array of drugs many of which are new, and many of the old ones are combined in new formulas with new and unfamiliar trade names that confuse the prescriber. Then again, the recent discoveries in the scientific world have an important bearing on modern therapeutics. Scarcely more than a decade has passed since Professor Roentgen discovered while experimenting with the cathode rays a peculiar and hitherto unknown radiative invisible to the naked eye, but capable of passing through substances opaque to ordinary light and of producing luminous effects when allowed to fall on fluorescent substances and like light, able to produce the chemical decomposition necessary for producing photographic effects.

The following year Henri Becquerel observed that the salts of uranium without previous exposure to sunlight were capable of giving off rays similar in their action to the Roentgen or *x*-

---

1. Read at the thirty-ninth annual meeting of the Medical Association of Central New York at Syracuse, October 16, 1906.

rays. A few months later the rare substances, polanium and radium, were discovered possessing radioactivity 100,000 times as great as uranium. Some theory had to be propounded to account for this strange phenomena known as radioactivity.

Dalton's theory of the indestructibility of atoms was shaken and a new theory was advanced by Professor Thomson and other physicists, who believe that it is possible to break off from an atom a quantity not exceeding the one one-thousandth of the whole. When a current of electricity is passed through a gas, it is supposed that some of the atoms are torn apart, divided into the so-called corpuscles, which carry opposite charges of electricity and so repel each other. This division of atoms is termed ionization of gases, the pet hobby of the electrotherapeutics.

Long ago when the spectroscope revealed to the scientist that, along with the evolution of the planets from their parent nebulae-there was a corresponding evolution of the chemical elements from simpler forms of matter, it dawned upon him that possibly matter is, in the final analysis, one *of these*. This suspicion has been greatly strengthened by the recently observed phenomena. However, the up-to-date radio-therapeutist has not attempted the direct realization of the alchemist's dream, but has contented himself with getting his gold (from the pockets of his patients) by the simple process of extraction.

Then again, the scientific psychology of the present and certain new and strange cults have their influence in the treatment of disease. Mystic medical doctrines have always had a following, from the days of Mesmer to the advent of Dowie and Mary Baker Eddy, and even members of the ethical profession claim to cure many diseases through suggestion and hypnotism. We must admit that modern therapeutics is somewhat encroached upon by the mystical.

Now, it is a well recognised principle that any great event, any important discovery or announcement, is accompanied by an unusual degree of susceptibility. This principle applies not only to individuals, families or nations, but to schools of thought and the professions as well. The medical profession is at present in this period of susceptibility, whereby it is easy to cut loose from the old and time worn paths and to rush headlong into the unknown realms of therapeutics. In the field of radioactivity, enthusiasm has certainly outrun caution. We have talked too much and promised too much. After all the excitement subsides, and we calmly and judiciously weigh the evidence it may appear that radioactivity is of little therapeutic value. Electricity, too, may not be the cure-all it is believed to be by its ardent devotees.

Commercialism, whether we are willing to admit it or not, is becoming a powerful if not a dominant influence in our profession. We do not have to look far beneath the surface to discover a restlessness, a secret admiration for a certain school of quacks who are making medicine a highly remunerative business and who curse the code and the ethical profession. Medicine is, or should be, more exalted than a mere business, but I am persuaded the crackling of electrical machines, the discharge of Leyden jars, the flashing of violet light and all the accompanying mystifying phenomena of the radio- and electrotherapeutist are in many instances the means whereby medicine is degraded to the level of a mere business. I do not desire to be understood as stating that all men who are using the various forms of mechanical treatment, are of necessity quacks. Far from it. But the use of these things for the purpose of getting money from the gullible public can not be too severely censured. I believe it is also true that the indiscriminate use of machines in therapeutics tends toward a lighter regard for medical ethics and the ethical profession.

Then again, physicians, and I take to myself a share of blame, are exceedingly susceptible to the wiles of the manufacturing chemist. His well dressed, smooth tongued and persuasive representative delights in unfolding to our wondrous gaze his spacious cabinets, filled with many colored samples of the present day pharmaceutical elegance and efficiency. As we think of it, we recall the old rhyme,

"Will you walk into my parlor,
Said the spider to the fly,
Its the nicest little parlor
That ever you did spy."

And we walk into the meshes of the pharmaceutical web he has woven for us and which he is holding up with ever increasing power. Now, how many of us can tell the exact ingredients with their relative proportion in all the pills and tablets we are using? Hands up! Time was when the doctor had a few remedies whose value he thoroughly knew and which he wisely employed in the treatment of many diseases, but now we have very many remedies for the treatment of almost every single ailment. It is so easy to dispense "so and so's" pills and tablets and elixirs, labeled for this and that complaint, that we are in danger of excessive drugging, drifting into polypharmacy with all its dire results and of losing our knowledge of individual drug action, which our medical forefathers possessed.

Is it not time we cut our drug bills a little for our own and our patient's benefit? Is it not time, also, that we aroused ourselves from our therapeutic laziness and began to do our own thinking in fitting carefully selected remedies to our cases, instead of allowing the manufacturing chemist do it for us in selecting the case for the remedy? The manufacturing chemist can do us much good. He can also do us much harm, if we allow him, and we must bear in mind that money is his object first, last, and all the time.

We have already alluded to the susceptibility we show towards Christian Science and allied doctrines, and only say in passing that the therapeutist who enters the field of medical mysticism is treading on dangerous ground, and unless our attempts to cure by suggestion and kindred methods are carefully guarded, we may be subjected to disappointment and chagrin.

But I must hasten to my closing thought—our duty. There have always been enthusiasts in medicine, exploiting this or that invention or doctrine. The future will continue to produce enthusiasts for, as it has been finely stated, "The history of the past will be the experience of the future." But I am persuaded that but little real good has been done the healing art by the enthusiasts. Progress in therapeutics has been rather by slow and patient endeavor and calm and deferred judgment,—"Line upon line, precept upon precept, here a little and there a little." So have we climbed and so must we continue to plod along our way. The history of medicine is full of the announcements of cures and systems of cures that have, in most instances, been relegated to the rubbish heap by the test of time. After all, we have few if any specifics. The practice of medicine is still difficult and oftentimes discouraging, but our best therapeutic friends are the old time honored drugs.

Radioactivity, vibration, electricity may aid a little, but can never replace drugs in the treatment of disease. It is our duty to seek a happy medium between therapeutic nihilism and polypharmacy, and to ever study the principles that govern the rational treatment of disease by drugs.

The new roads promise to lead us to glorious things by short and direct routes and we must not be so ultraconservative as to totally ignore them but, as yet, we can not safely leave the old highways, for along them we are ever guided by the milestones set up for us by the great men who have gone before,—Sydenham, Bichat, George B. Wood, Alonzo Clark, Austin Flint, J. Milner Fothergill and scores of others who along conservative lines have left their impress upon the art of curing disease.

We must turn a deaf ear to the unconscious quackery of the present that finds its expression in the lamentable lack of restraint which characterises our acceptance of the new and untried. We must find our inspiration in the love of good and honest work and not in the sordid service of Mammon. It rests largely with the *conservative* therapeutist to keep medicine above the level of a mere business, to save medicine from its greatest enemy, commercialism. The best doctor is altruistic rather than selfish, cautious rather than imaginative, not choosing the will-of-the-wisps that the public ever delight in, but determined to know the truth that shall make him free indeed. "Verily he shall not lose his reward."

